# Natural Resistance of *Leishmania infantum* to Miltefosine Contributes to the Low Efficacy in the Treatment of Visceral Leishmaniasis in Brazil

**DOI:** 10.4269/ajtmh.18-0949

**Published:** 2019-08-19

**Authors:** Juliana B. T. Carnielli, Renata Monti-Rocha, Dorcas Lamounier Costa, Aretha Molina Sesana, Laura N. N. Pansini, Marcela Segatto, Jeremy C. Mottram, Carlos Henrique Nery Costa, Sílvio F. G. Carvalho, Reynaldo Dietze

**Affiliations:** 1 Núcleo de Doenças Infecciosas, Universidade Federal do Espírito Santo, Vitória, Brazil;; 2 York Biomedical Research Institute, Department of Biology, University of York, United Kingdom;; 3 Instituto Natan Portella para Doenças Tropicais, Universidade Federal do Piauí, Teresina, Brazil;; 4 Centro de Ciências Biológicas e da Saúde, Universidade Estadual de Montes Claros, Montes Claros, Brazil;; 5 Global Health and Tropical Medicine, Instituto de Higiene e Medicina Tropical, Universidade NOVA de Lisboa, Lisbon, Portugal

## Abstract

In India, visceral leishmaniasis (VL) caused by *Leishmania donovani* has been successfully treated with miltefosine with a cure rate of > 90%. To assess the efficacy and safety of oral miltefosine against Brazilian VL, which is caused by *Leishmania infantum*, a phase II, open-label, dose-escalation study of oral miltefosine was conducted in children (aged 2–12 years) and adolescent-adults (aged 13–60 years). Definitive cure was assessed at a 6-month follow-up visit. The cure rate was only 42% (6 of 14 patients) with a recommended treatment of 28 days and 68% (19 of 28 patients) with an extended treatment of 42 days. The in vitro miltefosine susceptibility profile of intracellular amastigote stages of the pretreatment isolates, from cured and relapsed patients, showed a positive correlation with the clinical outcome. The IC_50_ mean (SEM) of eventual cures was 5.1 (0.4) µM, whereas that of eventual failures was 12.8 (1.9) µM (*P* = 0.0002). An IC_50_ above 8.0 µM predicts failure with 82% sensitivity and 100% specificity. The finding of *L. infantum* amastigotes resistant to miltefosine in isolates from patients who eventually failed treatment strongly suggests natural resistance to this drug, as miltefosine had never been used in Brazil before this trial was carried out.

## INTRODUCTION

American visceral leishmaniasis (VL), caused by *Leishmania infantum* (synonymous with *Leishmania chagasi* in Brazil), is a major health problem in many parts of Brazil. The disease is usually fatal if untreated and is characterized clinically by fever, gradual weight loss, splenomegaly, hypergammaglobulinemia, and pancytopenia.^1^

Visceral leishmaniasis treatment has been challenging because it relies on a few classic agents (pentavalent antimony, amphotericin B deoxycholate), all of which are parenteral and poorly tolerated. In Brazil, the standard therapeutic regimen is 20 mg Sb^v^/kg/day, for a minimum of 20 days. Amphotericin B deoxycholate is the second-line drug of choice. Lipid formulations of amphotericin B are less toxic but more expensive and, as a result, only available under request to the Ministry of Health for patients with severe disease with complications, for example, bleeding, and for children younger than 1 year and patients older than 50 years.^2^

In this scenario of scarcity of efficacious drugs, in the late 1990s, an oral drug, miltefosine (hexadecylphosphocholine), a phospholipid analogue, was considered an important advance in leishmaniasis therapy. Although developed originally as an anticancer drug, miltefosine is relatively safe and became the first-line therapy in India, where VL is caused by *Leishmania donovani*. The reported cured rates were high both in adults and in children, 94–95%.^3,4^ However, efficacy decreased after extensive use in the Indian subcontinent over the ensuing decade.^5–7^

There are no data on miltefosine effectiveness against VL caused by *L. infantum* in South America. This study was designed to evaluate the efficacy and safety of miltefosine in a phase II trial, in Brazilian patients with VL and investigate whether the clinical outcome could be associated with in vitro susceptibility of the parasites to miltefosine.

## MATERIALS AND METHODS

### Study design.

This was a phase II, open-label, dose-escalation study of oral miltefosine (Impavido^®^, supplied by AEterna Zentaris) in children (aged 2–12 years) and in adolescent-adults (aged 13–60 years) at two sites in Brazil, Montes Claros and Teresina. The objective was to investigate if efficacy and safety of oral miltefosine in Brazilian VL patients were similar to that already published for Indian VL patients.

### Patients.

The patients were enrolled and treated in 2005. Inclusion criteria were newly diagnosed (untreated) VL, with parasitological confirmation via visualization of amastigotes in tissue samples or a positive culture, and either gender. Exclusion criteria were severe decreases in the formed elements of the blood or host biochemical abnormalities: platelet count < 30 × 10^9^/L; white blood count (WBC) < 1 × 10^9^/L; hemoglobin < 5 g/100 mL; liver enzymes ≥ 3 times upper limit of normal range; serum creatinine or BUN ≥ 1.5 times upper limit of normal range. Other exclusion criteria were evidence of serious underlying disease (cardiac, renal, hepatic, or pulmonary); immunodeficiency or antibody to HIV; severe protein and/or caloric malnutrition (Kwashiorkor, Marasmus); any non-compensated or uncontrolled condition; and lactation, pregnancy (to be determined by adequate test), or inadequate contraception in females of childbearing potential for treatment period plus 2 months.

### Treatment.

The first patients were treated at Montes Claros with the recommended regimen of 2.5 mg miltefosine/kg/day, using the 10 mg formulation for 28 days. Adolescent-adults received 100 mg/day (one 50 mg capsule twice a day with meals), the same dose used in India. When cure rates for the 14 patients at Montes Claros were seen to be low, subsequent patients at Teresina were administered drug at the same daily dose (2.5 mg/kg/day for children, 100 mg/day for adults) for 42 days in an attempt to increase cure rates.

### Follow-up.

Patients were seen in follow-up at the end of treatment to ascertain initial cure and for 6 months after the end of therapy to ascertain final cure.

### Endpoints.

Failure was defined by clinical and parasitological criteria: signs or symptoms suggestive of leishmaniasis, accompanied by confirmation of the presence of *Leishmania* in a bone marrow aspirate at that time. Cure was lack of failure. The primary endpoint was efficacy: the rate of cure by the end of follow-up 6 months after treatment. Other endpoints were adverse events and correlation of in vitro susceptibility with cure.

### Sample size.

**Figure 1.**
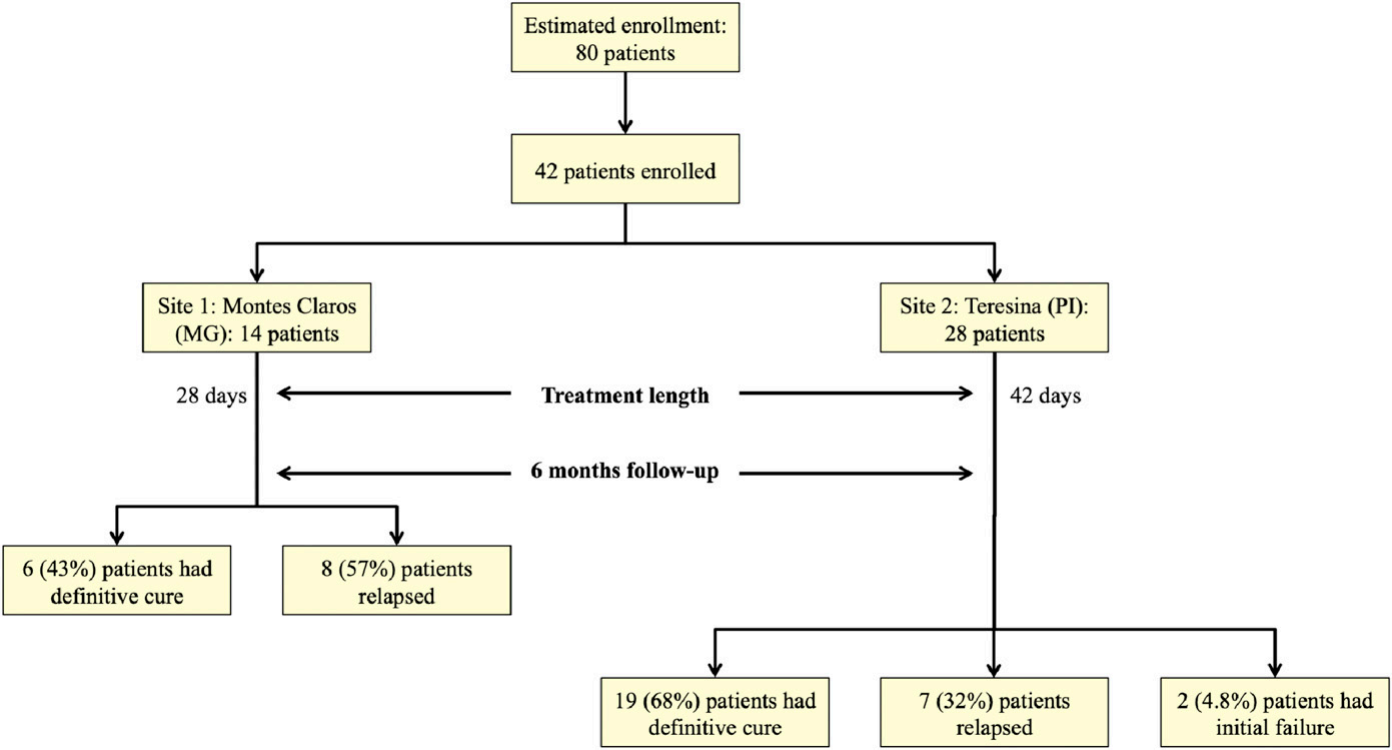
Flow charts of phase-II Brazilian miltefosine trial. This figure appears in color at www.ajtmh.org.

The sample size was chosen for convenience: up to 40 pediatric and 40 adolescent-adult patients (Figure 1).

### In vitro susceptibility of amastigotes to miltefosine.

*Leishmania* isolates from both sites were obtained before treatment by bone marrow aspiration from 26 of the final total of 40 patients. Promastigotes were grown in liver infusion tryptose (LIT) medium supplemented with 10% heat-inactivated fetal calf serum (Hi-FCS). All isolates were previously identified as *L. infantum* using polymerase chain reaction–restriction fragment length polymorphism.^8^ The miltefosine susceptibility of intracellular amastigote was performed as follows: adherent macrophages from peritoneal fluid of Swiss mice were infected with late-log phase promastigotes at a ratio of seven parasites to one macrophage, using the 16-well Lab-Tek tissue culture slides (Nunc, NY). After 24 hours of incubation in Roswell Park Memorial Institute (RPMI-1640) supplemented with 10% Hi-FCS in 5% CO_2_ at 37°C, free promastigotes were removed and the culture was then exposed to different concentrations of miltefosine (0, 0.55, 1.67, 5, and 15 µM) in triplicate. Higher concentrations were not tested because of drug toxicity to the infected macrophage. After 72 hours of additional incubation, the slides were stained with Diff-Quick solutions and 100 cells in each well were counted to determine the percentage of infected macrophages. Drug activity was determined from the percentage of infected cells in drug-treated cultures relative to nontreated cultures. The half-maximal inhibitory concentrations (IC_50_) of miltefosine for amastigotes were calculated from nonlinear regression analysis, using GraphPad Prism v.7.0a software (GraphPad Software, San Diego, CA). The results were expressed as the mean of three independent experiments. All assays were carried out blind regarding isolate identity and clinical outcome.

### Ethical review board.

Informed consent was obtained from all study participants and/or guardians before enrollment. The study protocol was reviewed and approved by the Institutional Review Board of the Universidade Estadual de Montes Claros, Universidade Federal do Piauí, and by the Brazilian National Review Board (CONEP D-18506-Z019). ClinicalTrials.gov Identifier: NCT00378495.

## RESULTS

### Montes Claros site.

Fourteen patients were enrolled in the clinical trial. Of these, 8 (57%), all children, failed to respond to treatment. The time of clinical and parasitological failure occurred at 1 month (one patient), 2 months (five patients), and 5 months (two patients) after the end of treatment (Figure 1). Laboratory and clinical data of cured and not cured patients are shown in Table 1. Adverse events were minor and included vomiting (four patients), nausea (three patients), and abdominal pain (two patients). These symptoms resolved when drug was given with the main meals.

**Table 1:** Entrance and end-of-treatment data

Site	1: Montes Claros		2: Teresina	
Treatment duration	28 days		42 days	
Parameter	Day 0*	Day 28*	Day 0*	Day 42*
Age (years)				
Cured	17 (2–43)	NA	19 (2–53)	NA
Failed	5 (2–11)	NA	17 (2–41)	NA
Duration of illness (days)				
Cured	28 (5–60)	NA	38 (9–60)	NA
Failed	32 (15–90)	NA	50 (21–120)	NA
Spleen size (cm)				
Cured	8.3 (4–15)	2.8 (0–4.5)	8.8 (2–14.5)	1.8 (0–5.5)
Failed	10.5 (7–12.5)	4.1 (2–4.5)	8.5 (0–12)	3.1 (0–7)
Hemoglobin (g/dL)				
Cured	8.8 (6.6–12)	11.6 (10–13.7)	8.5 (5.2–10.8)	11.5 (7.5–14.6)
Failed	8.3 (6.7–10.6)	10.0 (8.7–12.3)	8.4 (6–11)	11.3 (10.2–13.5)
WBC (×1,000/mm^3^)				
Cured	3.6 (1.8–7.3)	6.8 (2.7–14.4)	3.2 (1.3–8.9)	7.8 (2.4–15.2)
Failed	3.5 (2.7–5.3)	7.7 (6.1–12.9)	2.6 (1.6–3.6)	7.5 (3.6–11.8)
Albumin (g/dL)				
Cured	3.1 (2.6–3.9)	4.3 (3.7–4.9)	3.3 (2.6–3.9)	4.0 (3.5–4.8)
Failed	3.6 (3–4)	4.2 (3.9–4.9)	3.3 (2.4–4.3)	4.1 (3.6–4.8)

* Data represent mean (range) of values.

Laboratory reference values: hemoglobin, 12–17 g/dL; WBC, 5,000–10,000/mm^3^; albumin, 3.5–5.5 g/dL.

Because of the high failure rates for these first 14 patients, the protocol was amended for subsequent patients such that they received the same daily dose but for an extended period of 42 days. These subsequent patients were enrolled at the site in Teresina.

### Teresina site.

Thirty patients were enrolled. Four patients prematurely terminated treatment: one patient because HIV infection was detected and three patients because of drug toxicity. A 56-year-old male was removed on day 12 because of renal toxicity and suspected treatment failure; a 4-year-old female was removed on day 6 because of vomiting; a 26-year-old male was removed on day 12 because of liver toxicity and suspected treatment failure. Thus, 26 patients (12 children and 14 adolescent-adults) completed the 42 days of treatment of whom 7 (three children and four adolescent-adults) failed to respond to treatment. The time of clinical and parasitological failure was at 1 month (one patient), 3 months (three patients), 4 months (two patients), and 6 months (one patient) after the end of treatment. Considering all 28 evaluable patients (26 per protocol patients plus two patients who may have failed at the time they were removed for reasons of intolerance), there were nine failures (32%) consisting of three children and six adolescent-adults. If we use an intention-to-treat analysis, considering all 30 enrolled patients, there were 11 failures (36%). Laboratory and clinical data of cured and not cured patients are shown in Table 1 and Figure 1.

### Both sites.

A summary of the outcomes for all patients enrolled at both clinical sites is shown in Table 2. For children, extension of treatment period from 28 days (Montes Claros site) to 42 days (Teresina site) improved the cure rate from 43% to 67%, although with the small number of patients in this phase II study, this difference was not statistically significant (*P* = 0.0995: Fischer’s exact test). For adolescent-adults, the cure rate at the Teresina site, the only site with appreciable numbers of patients, was 69%. Inspection of the entrance characteristics does not reveal obvious differences between parameter values for patients destined to cure or to fail treatment with miltefosine (Table 1).

**Table 2:** Final efficacy data

	Children	Adolescent-adults	All ages
Montes Claros*	11	3	14
Cure	3 (27%)	3 (100%)	6
Failure	8 (73%)	0 (0%)	8
% Cure (95% CI)	27%	100%	43% (18–71)
Teresina†	12	16	28
Cure	8 (67%)	11 (69%)	19
Failure	4 (33%)	3 (19%)	7
Probable failure	0 (0%)	2 (12%)	2
% Cure (95% CI)	67%	69%	68% (48–84)

* Patients from Montes Claros were treated for 28 days.

† Patients from Teresina were treated for 42 days.

### Miltefosine susceptibility of *L. infantum* isolates.

**Figure 2.**
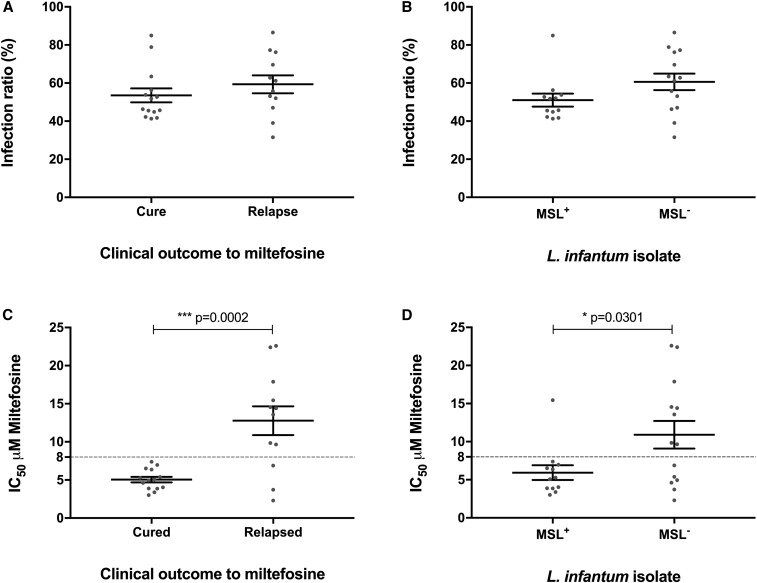
Infectivity and in vitro miltefosine susceptibility of *L. infantum* isolates collected before treatment for cured and failed patients. (**A**) and (**B**) Infection ratio, percentage of infected macrophage 96 hours post infection with *L. infantum* isolates. (**C**) and (**D**) In vitro miltefosine susceptibility of intracellular amastigote stage of *L. infantum* isolates. Each symbol represents the value for an individual *L. infantum* isolate. The horizontal bars in panels indicate the mean values for the groups and the standard errors of the means. Asterisk indicates significant difference between groups (*t*-test). MSL^−^ = homogeneous population for absence of MSL; MSL^+^ = homogeneous/heterogeneous population for presence of MSL.

To test if the differences in clinical outcome to miltefosine treatment could be explained by variance in miltefosine susceptibility of the infecting parasite *L. infantum*, the in vitro susceptibility of intracellular amastigote of *L. infantum* to miltefosine was assessed for pretreatment parasites from 26 patients: 14 parasite isolates came from patients who cured and 12 parasite isolates from patients who failed. There was no significant difference in *L. infantum* infectivity on a peritoneal mouse macrophage model when pretreatment isolates from cured patients and those who failed were compared (Figure 2A). Moreover, no differences were observed in *L. infantum* infectivity ratio between parasites containing or not the miltefosine sensitivity locus (MSL), which we previously associated with miltefosine treatment outcomes^9^ (Figure 2B). Miltefosine concentration that reduces 50% of infected macrophages (IC_50_) was calculated and the in vitro IC_50_ values of these pretreatment isolates showed a significant difference between isolates from patients who were cured (mean IC_50_ = 5.1 µM: SEM = 0.4 µM) and those who failed (mean IC_50_ = 12.8 µM: SEM = 1.9 µM): *P* = 0.0002 via *t*-test (Figure 2C). The in vitro susceptibility assay also showed a significant difference between isolates MSL^+^ (mean IC_50_ = 5.9 µM: SEM = 1.0 µM) and MSL^−^ (mean IC_50_ = 10.9 µM: SEM = 1.8 µM), which corroborates our previous association between MSL and treatment outcome (Figure 2D). By ROC curve analysis, the IC_50_ of 8.0 µM was highlighted as the best discrimination point to predict treatment outcome, such that isolates with lower IC_50_ would be predicted to cure and higher IC_50_ would be predicted to fail. Based on the IC_50_ values of the 26 *L. infantum* isolates, cure was predicted for 17 patients, but only 14 patients were cured. Among nine isolates for which failure was predicted, all patients relapsed, resulting in a sensitivity of 82% and specificity of 100%.

## DISCUSSION

The efficacy of miltefosine in the treatment of South American (Brazilian) VL was lower than expected. At the first site, where most patients were pediatric, miltefosine was administered for the standard 28 days, resulting in a cure rate of 43% (6 of 14 patients). Previous pharmacokinetic studies showed that miltefosine exposure was lower in pediatric patients compared with adult patients with VL, which has been linked with treatment failure.^10–12^ However, a comparable pediatric and adolescent-adult cure rate (67% and 69%, respectively) was observed at the second site, where an extended period of treatment (42 days) was performed. In a nonrandomized comparison, the extended treatment course improved slightly the cure rate compared with the 28-day standard regimen (68% or 64% in an intention to treat analysis). Nevertheless, this cure rate is far below the > 90% cure rate for miltefosine reported in Indian VL before its widespread use.^2–4,13^

Because the values of entrance clinical and laboratory parameters did not predict eventual cure and confounding concomitant diseases were ruled out by exclusion criteria, we hypothesized that inherent differences in parasite susceptibility to drug might be predictive of therapeutic success. To this end, we cultured parasites from 26 of the 40 enrolled patients, with approximately equal numbers of parasites from eventual cures (14 parasites) and failures (12 parasites). The in vitro miltefosine susceptibility phenotype of intracellular amastigotes showed that IC_50_ values of the pretreatment isolates were able to distinguish eventual cures from eventual failures, strongly suggesting that treatment failure observed in Brazil is associated with natural resistance of *L. infantum* parasite to miltefosine.

Killing of parasites by drugs depends on pharmacokinetics and pharmacodynamics, which may vary in different VL patients in different parts of the world. For Indian subcontinent VL, the lower rate of efficacy of miltefosine in children versus adolescent-adults^14^ may be linked to lower exposure of the drug in children.^15^ With respect to activity of drug against the parasite, Prajapati et al.^16^ found similar pretreatment IC_50_ in promastigotes from patients destined to cure (5.8 µM) compared with patients destined to fail (4.5 µM). However, there was a slight increase in parasite resistance to miltefosine as shown by an increase in IC_50_ posttreatment (6.1 µM) compared with pretreatment (3.7 µM). Bhandari et al.^17^ showed an increase in IC_50_ in isolates at the time of failure (4.7 µM) compared with pretreatment (1.9 µM), and Deep et al.^18^ confirmed that IC_50_ increase on failure (11 µM) compared with pretreatment (3.9 µM). Our present report appears unique in finding significant differences between the activity of miltefosine against VL pretreatment that correlate with eventual treatment outcome. Our findings are of particular interest because, as mentioned previously, the different susceptibility of *Leishmania* to miltefosine that correlates with clinical outcome is inherent to Brazilian *L. infantum* because these data from 2005 predates any use of this drug in Brazil.

Our recent genomic analysis showed a strong association (*P* = 0.0005) between the deletion of the *L. infantum* MSL and miltefosine treatment failure. The absence of MSL in the parasite increases the risk of treatment failure 9.4-fold and predicts miltefosine failure with 92% sensitivity and 78% specificity, which highlights MSL as a potential molecular marker to predict miltefosine treatment outcome in VL.^9^ Our data suggest that neither the presence nor absence of the MSL influences the ability of the parasites to infect macrophages in vitro. However, there is a strong correlation between the presence of the MSL and the in vitro susceptibility of amastigote stage to miltefosine (*P* = 0.0301). This result highlights that screening for the presence of the MSL by polymerase chain reaction could be a convenient prognostic marker in clinical practice to predict efficacy of miltefosine because it can be performed directly on biological samples in a much shorter time than the in vitro characterization of the susceptibility of the parasite to miltefosine. The molecular mechanism involved in the resistance of *L. infantum* to miltefosine in these strains, however, is still unknown and subject to further investigation.

Finally, some limitations of our study should be taken into account: (1) the lack of a randomized controlled trial can limit inferences from our results; (2) this trial was carried out at only two sites, one in the northeast and the other in the southeast part of Brazil; (3) the trial had to be interrupted before it reached the planned simple size, because of ethical reasons (low efficacy), resulting in only 44 patients enrolled; (4) the trial was split into two different dosing regimens. Moreover, considering that the *L. infantum* studied here were isolated over a decade ago, it is also important to know the miltefosine-resistant phenotype throughout Brazil now. We have started to address this point by screening for the MSL frequency in different areas of Brazil and found that MSL frequency changes geographically (data not shown), indicating that miltefosine could be successfully used after MSL stratification in some parts of Brazil.^9^
